# Special Issue “New Insights into the Effects of Microbiome-Derived Metabolites on Health and Disease”

**DOI:** 10.3390/ijms27104575

**Published:** 2026-05-20

**Authors:** Kinga Jaworska

**Affiliations:** Department of Experimental Physiology and Pathophysiology, Medical University of Warsaw, Banacha 1B, 02-097 Warsaw, Poland; kinga.jaworska@wum.edu.pl

## 1. Introduction

The human gastrointestinal tract is increasingly recognized not merely as a site of digestion, but as a fully integrated metabolic organ [1]. We have moved past the era of simply cataloging microbial taxonomy; the current frontier lies in functional metabolomics, the “chemical language” through which microbes communicate with their host [2]. These metabolites, ranging from short-chain fatty acids (SCFAs), methylamines, to neurotransmitters and modified bile acids, enter the systemic circulation and influence distant organ systems, including the heart, brain, and liver [3].

Since the launch of this Special Issue, the field has continued to evolve with remarkable speed. High-impact research has recently identified new metabolic drivers of chronic disease that underscore the clinical urgency of this topic. For example, large-scale studies have linked gut-processed sugar alcohols, such as xylitol, directly to increased cardiovascular risk and platelet reactivity [4]. Furthermore, our understanding of the gut–brain axis has been refined by discoveries showing how specific microbial metabolites, such as indole-3-propionic acid, act as neuroprotective agents that modulate neuroinflammation [5]. These few examples represent only a fraction of a rapidly expanding field, yet they perfectly complement the diverse contributions included in this collection.

This Special Issue, “New Insights into the Effects of Microbiome-Derived Metabolites on Health and Disease,” brings together five diverse contributions, two Original Articles and three Reviews, that highlight the multifaceted roles of the microbiome in pathophysiology, toxicology, and innovative therapeutic modeling.

## 2. Metabolic Disruptions and Toxicology

Environmental and dietary factors significantly influence the microbial metabolic landscape. The study by Jeong et al. (Contribution 1) provides a comprehensive subacute toxicity analysis of Deoxynivalenol (DON), a common mycotoxin found in cereal crops. Using a rat model, the authors demonstrate that DON contamination does not merely cause localized gut irritation but induces systemic changes in blood biochemistry and histology. Crucially, their metabolomic and microbiota profiling reveals that DON exposure alters the microbial community structure and metabolic output, suggesting that the toxicological profile of food contaminants is intimately linked to the disruption of the gut microbiome–metabolome interactions.

## 3. TMAO: A Diagnostic and Prognostic Dilemma

One of the most extensively studied microbiome-derived metabolites is Trimethylamine N-oxide (TMAO). In our review (Contribution 2), we explore TMAO as a multifaceted biomarker in cardiovascular disease. We highlight the “TMAO paradox”—where high levels are associated with adverse cardiac events, yet its role as a direct causative agent versus a bystander remains debated. Above all, TMAO should be viewed as a multifaceted biomarker that integrates dietary habits, microbial activity, and kidney function. We propose that future clinical applications must move toward personalized reference ranges that account for these individual metabolic variables.

## 4. The Gut–Brain Axis and Neuropsychiatric Health

Two contributions in this issue delve into the burgeoning field of the gut–brain axis, focusing on the intersection of genetics and microbial influence. Mihailovich et al. (Contribution 3) examine the “Microbiome–Genetics Axis” in Autism Spectrum Disorders (ASD). Their review emphasizes how probiotics may modulate neurodevelopmental outcomes by altering the metabolic environment of the gut, providing a hopeful perspective on non-invasive dietary interventions for complex genetic disorders. The study emphasizes that microbial metabolites act as epigenetic modulators that can influence the expression of ASD-related genes. Their findings suggest that specific probiotic strains do more than just improve gastrointestinal symptoms; they can potentially normalize metabolic signatures, such as the ratio of p-cresol to other phenols, that are often dysregulated in ASD patients. This provides a compelling argument for metabolic-based “psychobiotics” in neurodevelopmental therapy.

To advance our mechanistic understanding of these interactions, Mihailovich et al. (Contribution 4) also present a cutting-edge review on the use of induced pluripotent stem cell (iPSC)-based approaches. They argue that traditional animal models often fail to replicate the nuanced chemical signaling of the human gut–brain axis. By creating sophisticated in vitro models, researchers can now study host–microbe interactions in neuropsychiatric disorders with unprecedented precision. This paper highlights how these “human-on-a-chip” or organoid technologies are essential for validating the roles of specific microbiome-derived metabolites in human neural signaling.

## 5. Intestinal Homeostasis and Oncogenesis

Finally, the local effects of the microbial environment on the intestinal epithelium are explored by Magnusson et al. (Contribution 5). By exposing colon-derived epithelial monolayers to fecal luminal factors from patients with Colorectal Cancer (CRC) and Ulcerative Colitis, the authors identified distinct gene expression patterns triggered by the specific metabolic and chemical “cocktails” present in these disease states. Interestingly, while both “cocktails” triggered inflammatory pathways, the CRC-derived factors specifically upregulated genes associated with cell proliferation and DNA damage response. This leads to the crucial conclusion that the microbiome-derived metabolome in the colon is a primary driver of the transition from chronic inflammation to oncogenesis.

## 6. Concluding Remarks

The articles collected in this Special Issue illustrate that microbiome-derived metabolites are not merely byproducts of digestion; they are potent signaling molecules that dictate health and disease states. From the toxicity of our food supply to the complex signaling pathways of the brain and heart, the “metabolic fingerprint” of our microbiota is central to modern molecular medicine. As we move forward, the integration of multi-omics data with advanced human-based modeling systems (such as iPSCs) will be vital for translating these insights into targeted clinical therapies.

## 7. Future Directions

Several key trajectories for research and clinical translation emerge from the findings of this Special Issue. First, there is a pressing need for the standardization of “metabolic biotypes” to establish population-specific baselines for key signaling molecules. As highlighted by the studies on TMAO and ASD, moving beyond binary “presence or absence” toward defining precise therapeutic windows for metabolites will be essential for the next generation of clinical diagnostics.

This diagnostic precision must be matched by experimental rigor, specifically through the integration of iPSC-derived organoids with microbial metabolomics. These high-fidelity human models represent a critical frontier, as they allow us to bypass the limitations of animal models and perform high-throughput screening of “metabolic correctors.” Whether through small molecules or the engineering of specific bacterial strains, the goal is to develop therapies that can actively restore a homeostatic metabolic profile in patients struggling with chronic inflammatory or neuropsychiatric conditions. Furthermore, the impact of environmental contaminants like DON on microbial health underscores the necessity of including the microbiome–metabolome as a standard endpoint in future toxicological assessments. Ultimately, protecting the metabolic output of our commensal microorganisms may prove as vital as protecting host organs from environmental toxins.

As Guest Editor, I would like to thank the authors for their high-quality contributions and the reviewers for their rigorous evaluation. I hope this collection serves as a catalyst for future research that eventually translates these molecular insights into bedside therapies.

## Abbreviations

The following abbreviations are used in this manuscript:

ASD	Autism Spectrum Disorders
CRC	Colorectal Cancer
DON	Deoxynivalenol
iPSC	induced pluripotent stem cell
TMAO	Trimethylamine N-oxide
